# Supplementary material for: Antimicrobial Effects and Antioxidant Activity of Myrtus communis L. Essential Oil in Beef Stored under Different Packaging Conditions
Source: Foods. 2023 Sep 10;12(18):3390. doi: 10.3390/foods12183390 (PMC10529660; doi:10.3390/foods12183390)
Supplement: Supplementary file 1 [file foods-12-03390-s001.zip › foods-2549242-supplementary.pdf]

*Supplementary file*

Table S1. Volatile compounds *of Myrtus Communis* L. Essential oil determined by GC-MS.

| RT (min) | KI | Compounds | Quantity (%) |
|---|---|---|---|
| 7.40 | 851 | 2-Hexenal | 0.7 |
| 8.25 | 900 | Nonane | 0.3 |
| 8.49 | 902 | Propanoic acid, 2-methyl-, 2-methylpropyl ester | 1.8 |
| 9.10 | 940 | α-Pinene | 4.4 |
| 10.03 | 981 | β-Myrcene | 0.8 |
| 10.22 | 991 | Isobutyl 2-methylbutanoate | 0.3 |
| 10.46 | 1003 | Propanoic acid, 2-methyl-, butyl ester | 0.7 |
| 10.92 | 1030 | Limonene | 6.2 |
| 11.00 | 1033 | Eucalyptol (1,8-cineole) | 9.9 |
| 12.10 | 1098 | β-Linalool | 12.3 |
| 13.65 | 1179 | Terpinen-4-ol | 0.6 |
| 13.86 | 1191 | α-Terpineol | 2.7 |
| 14.00 | 1200 | Myrtenol | 1.2 |
| 14.34 | 1229 | Nerol | 0.5 |
| 14.72 | 1241 | Linalyl o-aminobenzoate | 5.9 |
| 15.63 | 1297 | trans-Pinocarvyl acetate | 1.2 |
| 15.87 | 1319 | 2,6-Octadienoic acid, 3,7-dimethyl-, methyl ester | 0.3 |
| 16.05 | 1325 | Myrtenyl acetate | 15.5 |
| 16.27 | 1339 | 2-Oxabicyclo[2.2.2]octan-6-ol, 1,3,3-trimethyl-, acetate | 0.3 |
| 16.37 | 1343 | α-terpinyl acetate | 2.2 |
| 16.69 | 1383 | Geranyl acetate | 7.4 |
| 17.09 | 1401 | Methyleugenol | 1.8 |
| 17.26 | 1409 | p-Menta-1(7),8-dien-9-ol | 0.2 |
| 17.61 | 1418 | Ethyl dihydrocoumarin | 0.3 |
| 17.72 | 1427 | β-caryophyllene | 0.4 |
| 18.11 | 1052 | 6,6-dimethylbicyclo [3.1.1] hept-2-en-2-yl, methyl-ethyl-carbonate | 0.4 |
| 18.22 | 1447 | Humulene | 0.9 |
| 18.93 | 1453 | 7-Isopropyl-7-methyl-nona-3,5-diene-2,8-dione | 1.7 |
| 19.21 | 1608 | Ethanone, 1,1'-(5-hydroxy-2,2-dimethylbicyclo[4.1.0]heptane-1,7-diyl)bis- | 0.3 |
| 20.07 | 1609 | Caryophyllene oxide | 0.7 |
| 20.40 | 1610 | Humulene-1,2-epoxide | 1.2 |

| | | | |
|---|---|---|---|
| 20.67 | 1631 | Diepicedrene-1-oxide | 0.3 |
| | | **Total** | **83.3** |

RT- Retention time; KI- Kovats index
